# Evaluation of Human Gingival Fibroblasts (HGFs) Behavior on Innovative Laser Colored Titanium Surfaces

**DOI:** 10.3390/ma16134530

**Published:** 2023-06-22

**Authors:** Susi Zara, Giulia Fioravanti, Angelo Ciuffreda, Ciro Annicchiarico, Raimondo Quaresima, Filiberto Mastrangelo

**Affiliations:** 1 Department of Pharmacy, University G. D’Annunzio of Chieti-Pescara, 66100 Chieti, Italy; susi.zara@unich.it; 2 Department of Physical and Chemical Sciences, University of L’Aquila, 67100 L’Aquila, Italy; giulia.fioravanti@univaq.it; 3 Clinical and Experimental Medicine Department, University of Foggia, 71122 Foggia, Italy; angelo_ciuffreda.555104@unifg.it; 4 Independent Researcher, Bari 70124, Italy; annicchiarico.ciro63@gmail.com; 5 Department of Civil, Construction-Architectural and Environmental Engineering, University of L’Aquila, 67100 L’Aquila, Italy; raimondo.quaresima@univaq.it

**Keywords:** Human Gingival Fibroblasts, colored titanium surfaces, laser-induced coloration, biocompatibility, surface free energy, wettability

## Abstract

The use of ytterbium laser to obtain colored titanium surfaces is a suitable strategy to improve the aesthetic soft tissue results and reduce implant failures in oral rehabilitation. To investigate the relationship between novel laser-colored surfaces and peri-implant soft tissues, Human Gingival Fibroblasts (HGFs) were cultured onto 12 colored titanium grade 1 light fuchsia, dark fuchsia, light gold, and dark gold disks and their viability (MTT Assay), cytotoxicity (lactate dehydrogenase release), and collagen I secretion were compared to the machined surface used as control. Optical and electronic microscopies showed a HGF growth directly correlated to the roughness and wettability of the colored surfaces. A higher viability percentage on dark fuchsia (125%) light gold (122%), and dark gold (119%) samples with respect to the machined surface (100%) was recorded. All specimens showed a statistically significant reduction of LDH release compared to the machined surface. Additionally, a higher collagen type I secretion, responsible for an improved adhesion process, in light fuchsia (3.95 μg/mL) and dark gold (3.61 μg/mL) compared to the machined surface (3.59 μg) was recorded. The in vitro results confirmed the innovative physical titanium improvements due to laser treatment and represent interesting perspectives of innovation in order to ameliorate aesthetic dental implant performance and to obtain more predictable osteo and perio-osteointegration long term implant prognosis.

## 1. Introduction

According to the Oral Health Program of the WHO, oral diseases result in a major health burden for many countries and affect approximately 3.5 billion people worldwide throughout their lifetime [1].

It is estimated that oral health conditions treatment is expensive and usually not part of universal health coverage (UHC) [2].

Nowadays, in developing countries, tooth loss is considered a negative condition with social and psychological as well as functional implications associated with self-image loss and a reduction of the quality of life [3].

Among the most frequent conditions of oral disorders, it is possible to include edentulism. The results of the Global Burden of Disease Study 2010 showed a constant decrease in DALY rates for edentulism in the population standardized by age (Disability Adjusted Life Year), from 144/100,000 in 1990 to 89/100,000 [4], which is related to the development of osseointegrated implantology in the last thirty years [5].

The rapid evolution of dental implantology has made maxillary edentulism solvable in a predictable way [6].

However, even after osseointegration of the implant, there are several clinical conditions, due to a thin gingival biotype of the patient, such as gingival retraction, mucositis, or peri-implantitis, especially in aesthetic areas of the maxilla, which are responsible for showing a portion of the implant screw or the titanium prosthetic neck of the abutment [7].

These conditions of aesthetic failure of implant rehabilitation are increasingly frequent, representing an important limitation in long-term rehabilitation success, and are related to the specific color characteristics of the titanium screw currently on the market [8].

Furthermore, the accepted criteria for implant success are defined by Branemark and Albrecktsson predicted a physiologic marginal bone loss (MBL) of 1–1.5 mm around dental implants during the first year after loading, and <0.2 mm annually thereafter [9,10,11].

Recently, several technologies and treatments have been developed for the production of coloured titanium surfaces, such as electrodeposition [12] and the zirconia ceramic implants, which seem to demonstrate the best results in terms of the color panel.

However, the color electrodeposition or the zirconia ceramic implants produced smooth surfaces, which seem to demonstrate inadequate results in terms of soft and hard tissue cell adhesion and titanium osseo- and perio-integration [8,13].

In 2009, Pae et al. [14] showed comparable biological responses of Human Gingival fibroblasts (HGFs) on titanium and zirconia surfaces, and Matthes et al. [15] concluded that plasma treatment supported cell covering on titanium and zirconia disks and both abutment surfaces. Currently, few scientific studies have been promoted to evaluate the color of implant surfaces and abutments. In 2022, Bass [12] and Seyidaliyeva [16] showed interesting results on the titanium color palette, comparing different surfaces after anodizing treatment with zirconia surfaces.

However, recently, Mastrangelo F. et al. showed how the laser use applied to modify titanium surfaces have obtained a wide panel of colors and creates specific micro-roughness surfaces able to promote osseo- and perio-integration [8].

Currently, the scientific literature recommends rough implant surfaces because they are able to promote osseointegration with a high percentage of success [17,18,19].

However, to favor an adequate aesthetic response in implantology it would be interesting to produce colored titanium surfaces, and, currently, the laser treatment with ytterbium seems to be the only alternative treatment to the anodization process, which, however, produces smooth surfaces, or to the use of abutments or zirconia implants.

The role of surface, physical, and chemical properties has been thoroughly evaluated in soft and hard tissue integration. Wettability, roughness, and biological response in dental implant clinical evaluation is receiving increased interest and is still under investigation [20]. Surface wettability, hydrophilicity, and roughness can affect adsorption of proteins onto the surface, cell adhesions, bacterial adhesion and subsequent biofilm formation, and the rate of osseointegration in vivo [21]. Different chemical, thermal, and electrochemical methods have been used to produce different titanium surfaces’ properties and colors by means of the formation of a Ti oxide film in order to achieve osseointegration properties [22]. Within the different coloring methods used for aesthetic colored coatings or layers, the use of chemicals could produce allergic reactions, while the use of heat treatments could generate a uniform and non-reproducible color with low resistance to corrosion [23,24]. Furthermore, some studies have shown that the use of anodic oxidation could favor the color formation of the abutments, giving a better gingival aesthetic response [25], as well as increase the thickness of the oxide layer [26], thus obtaining a better resistance to surface corrosion.

However, anodic coloring has several disadvantages in terms of environmental and working risk, also producing smooth surfaces [27], which are not very suitable for some bioprocesses such as, for example, osseointegration [13,28]. It has been demonstrated that innovative and alternative titanium laser treatments having various colors are possible and able to produce a complete palette of colors [8] with different optical and physical features [13,23,24,29,30]. The coloring procedure itself is simple and based on the adopted laser power. No contaminants are induced on the native cp Ti surface as demonstrated by the EDX measures and there are no porous or marked grooves and pits. The novel laser coloring technique is ecofriendly, highly replicable, cheap, and cell compatible. Surface laser irradiation is able to modify all those chemical (oxide layer and periodic), physical (wettability), and mechanical (topography and roughness) features capable of positively improving cell adhesion, proliferation, and viability [31,32,33,34,35,36,37]. Even technologically simple [33], laser surface modification is a very complex process depending on many correlated parameters (speed and number of scans, energy density, spot size and variation of focal plane, pulse mode and repetition rate, power, fluency) [20,38]. Finished machined titanium surfaces have a roughness of micrometers, while in this case, the turning manufacturing produces a macro roughness of hundreds of nanometers. On the colored surface, compared with the machined one, the parameters, under patent and with a low power with high repetition rate, did not increase the roughness [34,39] and they did not produce any significant variation of the topography (pits, craters, or grooves) [40,41]. At high magnification, the electron microscopy shows that all the colored titanium surfaces produced appeared melted and cooled [42].

At atmospheric pressure, the different oxygen diffusions on the laser irradiated melted surface produce oxidized nanometer layers [33,35] able to interfere with the light and produce the colored aspect [8]. To titanium oxides and their thickness, authors attribute the absorption of albumin and fibrinogen blood proteins, which influences cell osseointegration or growth [33,43,44]. In order to better understand the behaviour of HGFs cultured on textured titanium surfaces, we deepened the analysis on the aforementioned materials by firstly performing a wettability test through the contact angle measurements. The liquid wets the surface of a solid by maximizing its area in contact.

The aim of the present in vitro study is to evaluate the behavior of primary HGFs cultured on different ytterbium laser-stained surfaces compared to smooth titanium surfaces.

## 2. Materials and Methods

A total of 15 commercial pure titanium (CpTi) Grade 1 disks (10 mm in diameter 0.5 thick) (Titanium Alloy-EuropaAcciai, Chieti, Italy) mechanically obtained by turning were ultrasonically cleaned (Elma Elmasonic S 60/H Gottlieb-Daimler-Straße 17 78224 Singen) in acetone (Carlo Erba, Milan, Italy) followed by Millipore water for 10 min for each step) and dried in a thermostatic oven (20 °C for 2 h). In a cleaning chamber, using an ytterbium laser with different selected sequences of nano-second pulses, the titanium surfaces were treated. Among all the colored titanium specimens obtained only light fuchsia, dark fuchsia, light gold, and dark gold colors were selected for the study. The laser-colored surfaces were compared to the titanium machined surface turning obtained.

### 2.1. Surface Analysis

The surface topographic features were analyzed by stereo (AXIO ZOOM, V16 Zeiss, Jena, Germany) and optical microscopy (Nikon Optiphot2, Tokyo, Japan). The surface color analysis was performed by spectrophotometry (X-Rite SP64, X-Rite Inc., Grand Rapids, MI, USA) with a diffuse illumination integrating sphere system (illuminant and reference angle for the output values of D50 2°). Colorimetric coordinates were expressed in the CIELAB standard colorimetric space defined by the International Commission on Illumination as lightness (L*) ranging from black (0) to white (100), as green (−) to red (+) (a*) and as blue (−) to yellow (+) (b*). The CIELAB system is able to define the numerical change in the Lab values and allows having a color measure that approximates human subjective vision (L* = 0 yields black and L* = 100 indicates diffuse white; specular white may be higher); values between red and green defined by a* (negative values indicate green, while positive values indicate red), between yellow and blue by b* (negative values indicate blue and positive values yellow).

The color variations between colored surfaces were detected by the CIELAB recommendations as (Equation (1)):

(1) $${∆E}_{\mathrm{ab}}^{*}=\sqrt{{\left(L_{2}^{*}-L_{1}^{*}\right)}^{2}+{{\left(a_{2}^{*}-a_{1}^{*}\right)}^{2}+\left(b_{2}^{*}-b_{1}^{*}\right)}^{2}}$$

### 2.2. Contact Angle Measurements

For wetting analysis, the static contact angle (CA) of liquids on surfaces was measured using a contact angle analyzer (Kruss, DSA100, Kehl, Germany). Contact angles (CA) of distilled water (polar) and diiodomethane (Prod. 158429, Sigma Aldrich, Merck, St. Louis, MO, USA, 99%) (apolar) liquids were measured both on Ti surfaces, to examine the wettability of surfaces by liquids with different surface energy components. For CA measurement, 2.0 μL of liquid drops was placed on a surface, and CA was measured within 1 s after the droplet settlement. After each measurement, the surfaces were cleaned with isopropyl alcohol (Prod. W292907, Sigma Aldrich, Merck, 99.7%) and dried with compressed air. At least three measurements were obtained from different spots of the surfaces, and the mean value was used for analysis.

When a liquid is placed on a solid surface there can be two cases: the liquid can spread to form a continuous film or form discrete droplets or, in the second case, the drop on the three-phase contact line, where the solid (S), the liquid (L), and the vapor (V) meet, can generate an angle called contact angle (θ). A range of different contact angle values can be observed, from 0° (called perfect wetting and, hence, spontaneous spreading) up to 180° (perfectly non-wetted surface). Water contact angles lower than 90° indicate hydrophilic surfaces, where wetting is favourable and the fluid spreads over a large surface area. Wetting phenomena on a macroscopic scale can be illustrated using Young’s Equation (Equation (2)).

(2) $$\gamma_{SV}=\gamma_{SL}+\gamma_{LV}cos\theta$$

where θ is the contact angle, γSV is the solid surface free energy, γSL is the solid/liquid interfacial free energy, and γ_LV_ is the liquid/vapor interfacial tension (liquid surface tension). Contact angle measurements of a surface using a probe liquid provides information about its wettability and Surface Free Energy (SFE). The SFE of a solid surface can provide information about how different liquids will interact with said surface and can be calculated using several models including the Owens–Wendt–Rabel–Kaelble (OWRK) model [45]. In the OWRK model, the solid SFE, γ_SV_, and the liquid surface tension, γ_LV_, are a sum of dispersive (e.g., London dispersion forces) and polar (e.g., hydrogen bonding) components, such that γ = γ^d^ + γ^p^. Then, the relationship between liquid surface tension, solid SFE, and the contact angle between the liquid and the solid is described by the OWRK Equation (Equation (3)):

(3) $$\gamma_{\mathrm{LV}}\left(1+\cos\theta \;\right)=2\;\left[{\left(\gamma_{\mathrm{SV}}^{d}\gamma_{\mathrm{LV}}^{d}\right)}^{1/2}+{\left(\gamma_{\mathrm{SV}}^{p}\gamma_{\mathrm{LV}}^{p}\right)}^{1/2}\right]$$

where θ is the contact angle between the liquid and the solid. At least two liquids with well-defined characteristics are required in the OWRK method, as there are two unknowns (solid/liquid interfacial free energy and solid surface free energy) that need to be solved to determine the solid surface free energy. At least one of the two liquids must have a polar part in addition to the disperse one. Water and diiodomethane are most often utilized. Distilled water is a highly polar liquid as its polar component is 51.0 mN/m with total liquid surface tension of 72.8 mN/m [46]. Diiodomethane has only dispersive contribution (apolar solvent), with a value of total liquid surface tension of 50.8 mN/m [46].

### 2.3. Profilometer Analysis

Ti surfaces were analysed using a Dektak 6M Surface Profiler (Veeco Instruments, Inc., Plainview, NY, USA). The stylus radius was 12.5 µm, force of 3.0 mg. Measurements of 2 mm length along the North-South and West-East directions were performed back and forward in triplicate.

### 2.4. Scanning Electron Microscopy (SEM) and Energy-Dispersive X-ray Spectroscopy (EDX) Analysis

Scanning electron microscopy analysis (Gemini 5000, Zeiss, Germany) of the light fuchsia surface after HGF culture was performed. Chemical surface composition of Ti machined surface and light fuchsia was assessed by Energy-dispersive X-ray spectroscopy (EDX) (Atzec Live, High Wycombe, Oxford, UK) and Ultim Max 100 detector (Oxford Instruments, High Wycombe Buckinghamshire, UK) at a voltage of 20 kV. Cells were fixed in glutaraldehyde 2.5% in 0.1 M Cacodylate Buffer, pH 7.2, for 20 min at 4 °C. Then, samples were subjected to 3 washes in the cacodylate buffer and subsequently dehydrated with increasing alcohol solutions (25–50–70–90–100% EtOH). Final drying was performed by two passages of 15 min each in hexamethyldisilazane (HSMDA), and then left overnight in a fume hood to allow complete reagent evaporation.

### 2.5. Cell Culture

The project has obtained the approval of the Local Ethics Committee of the University of Chieti (approval number 1173, approval date 31 March 2016), in accordance with the Declaration of Helsinki. HGFs were isolated from gingival tissue fragments, as already reported elsewhere [47]. Titanium disks were sterilized with an UV lamp (1 h for each side) and placed in a sterile 48-well plate not treated for cell culture. Then, 20,000 cells were seeded on each titanium disc and cultured for 48 h. After 48 h, the culture supernatants were collected and frozen at −80 °C for subsequent analyses.

### 2.6. MTT Assay

The cell viability was evaluated after 48 h of culture by MTT (3-[4,5-dimethyl- thiazol-2-yl-]-2,5-diphenyl tetrazolium bromide) assay (Merck, Darmstadt, Germany), based on the ability of the mitochondrial dehydrogenase of viable cells to transform MTT into colored formazan salts. At established time points, the medium was replaced with a fresh one containing 0.4 mg/mL MTT, and the cells were incubated for 5 h at 37 °C. After a further incubation of the samples in DMSO for 20 min at 37 °C, 200 μL of each medium were transferred into a 96-well plate, and the absorbance was measured at 570 nm wavelength using a Multiscan GO microplate spectrophotometer (Thermo Fisher Scientific, Waltham, MA, USA). The values obtained without cells were subtracted from the values obtained from the samples. Three independent experiments were performed under the same experimental conditions.

### 2.7. Lactate Dehydrogenase (LDH) Cytotoxicity Assay

For the purpose of quantifying the cytotoxic effect exerted on HGFs by titanium discs, the CytoTox 96 Non-Radioactive Assay (Promega Corporation, Fitchburg, WI, USA) was performed. The assay quantitatively measures LDH release within the culture medium. Supernatants (50 μL) were pipetted in a 96 well plate with a flat bottom (Falcon, Corning Incorporated, New York, NY, USA) and the volume was doubled adding the LDH reaction mixture. After 30 min of incubation at room temperature in the dark, 50 μL of stop solution were added and the absorbance was measured at 490 and 690 nm wavelength; the values obtained were normalized on MTT values.

### 2.8. Collagen Type I (Col I) ELISA

Collagen type I released by HGFs, cultured on the aforementioned titanium surfaces, in the culture medium was evaluated using a Human Collagen Type 1 ELISA kit (Cosmo Bio Co., Ltd., Tokyo, Japan) following the manufacturer instructions. The absorbance was spectrophotometrically measured at 450 nm wavelength by Multiskan GO (Thermo Scientific, Waltham, MA, USA). The concentration of Col I (μg/mL) was calculated using a standard curve generated with specific standards provided by the manufacturer and normalized with the MTT values.

### 2.9. Statistical Analysis

Statistical analysis was carried out by one-way ANOVA, through Prism 5.0 software (GraphPad) followed by Tukey’s post-hoc test. Statistically significant values were established for *p* < 0.05.

## 3. Results

### 3.1. Optical Microscopy

**Figure 1:**
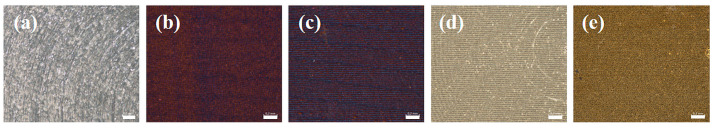
Aspect at the stereomicroscope of the Ti surfaces: (**a**) Machined, (**b**) light fuchsia, (**c**) dark fuchsia, (**d**) light gold, and (**e**) dark gold. Scale bar for all the surfaces is 0.2 mm.

The titanium surfaces were observed by stereomicroscope, as reported in Figure 1. All the surfaces are characterized by typical circular tracks produced by the turning working. Colors are produced by powered micro pulses applied through laser scanning. This operation produces thin layers of titanium oxide able to interfere with the light and generating the visible color [8].

The titanium machined surface, shown in Figure 1a, is characterized by typical circular tracks produced by the turning working. By increasing the applied power laser it is possible to produce higher titanium oxidation with a shifting from, respectively, Fuchsia to Gold and from light to dark hue (Figure 1b–e). The direction of the laser scanning is clearly visible on the surface (Figure 1c,d) while in the case of the gold color, the turning finishing is still observable (Figure 1d) or not (Figure 1e). Colorimetric coordinates were expressed in the CIELAB standard colorimetric space defined by the International Commission on Illumination. In Table 1, the three values of the CIELAB color space for Ti surfaces are reported. L* represents lightness from black to white on a scale of zero to 100, while a* and b* represent chromaticity with no specific numeric limits.

### 3.2. Contact Angle

**Figure 2:**
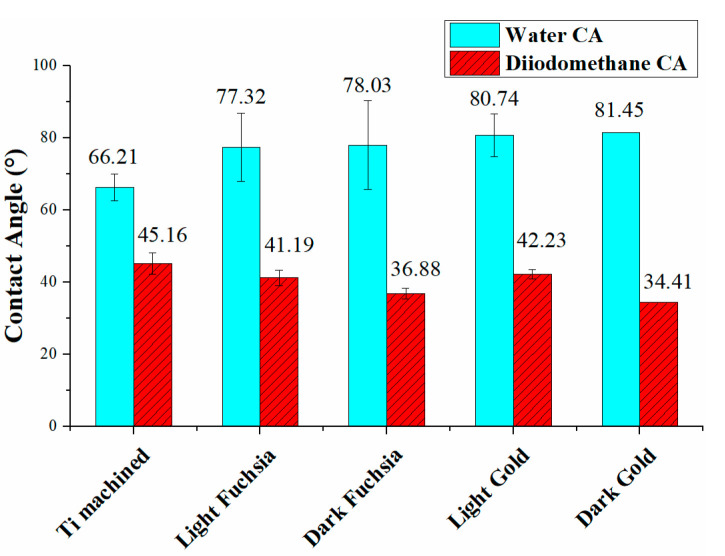
Water and diiodomethane Contact Angle measurement on Ti surfaces.

Figure 2 shows the water and diiodomethane CA for all the Ti surfaces. The contact angle increases from a value of about 66.21° for the machined Ti surface up to 81.45° for the dark gold surface.

**Figure 3:**
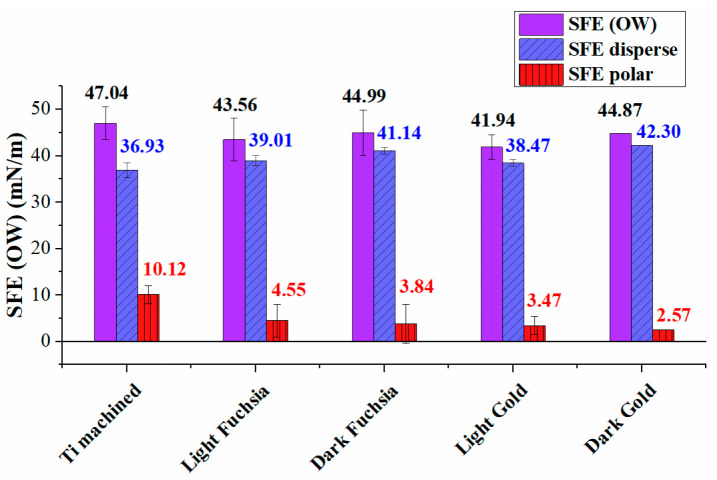
Surface Free Energy analysis for Ti surfaces (violet). Disperse (blue) and polar (red) components are also reported.

Figure 3 shows the polar and dispersive components of the surface free energy, calculated using the Owens Wendt approach, and the total SFE for all Ti surfaces. To better highlight the different components, a histogram graph was chosen, where the dispersive contributions are shown in blue, the polar contributions in red, and the total SFE in violet.

As observed in Figure 3, the overall SFE of laser-treated Ti surfaces is lower than the machined one, and the polar component has decreased, a trend most evident in the dark gold surface. The total SFE appears to have the same order of magnitude for all treated samples, considering the experimental errors.

Table 2 reports the values of the contact angles with the two selected solvents (water and diiodomethane), the dispersed and polar components of the surface energies, and the total SFE. The polar ratio is also shown for all samples, i.e., the ratio of the polar component to the total SFE.

The small differences between the SFEs are explained by the presence of a different polar component from surface to surface, the dispersive component being almost the same for all laser colored samples. All colored surfaces show higher SFE values than the machined surface. The ratio of polarity to the overall surface energy of solids was calculated, and reported in this table. The polar ratio decreased from 21.5% for the machined Ti surface to 5.7% of the dark gold surface.

### 3.3. Wetting Envelope

Liquid surface tension, solid SFE, and the contact angle a liquid droplet makes on the surface are all related, and their relationship can be visualized by the Wetting Envelope plot.

**Figure 4:**
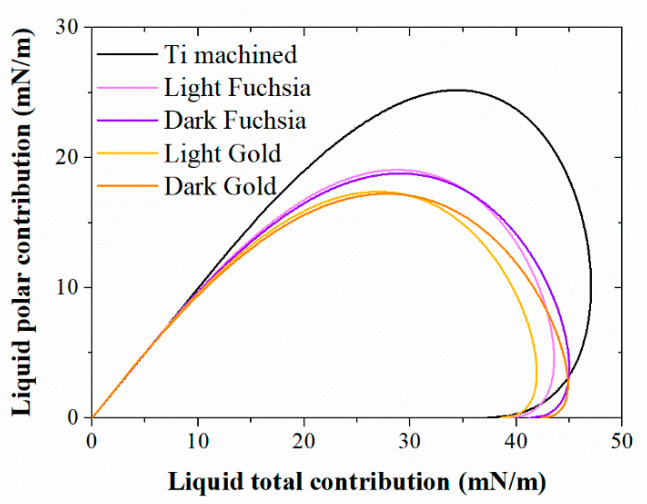
Wetting Envelope for Ti surfaces for complete wetting (θ = 0). In the horizontal axis is the total surface tension of liquids γ_L_ and in the vertical axis is the polar surface tension component γ_Lp_.

The polar and dispersive components are calculated using the Owens–Wendt equation and to highlight the differences between the various surfaces in terms of wettability, the polar contribution is plotted against the total surface tension in Figure 4. The envelope created for the case of a contact angle of 0° (complete wetting) is shown. All liquids whose surface tension falls below the wetting envelope have a high tendency to wet the solid surface.

### 3.4. Profilometer

**Figure 5:**
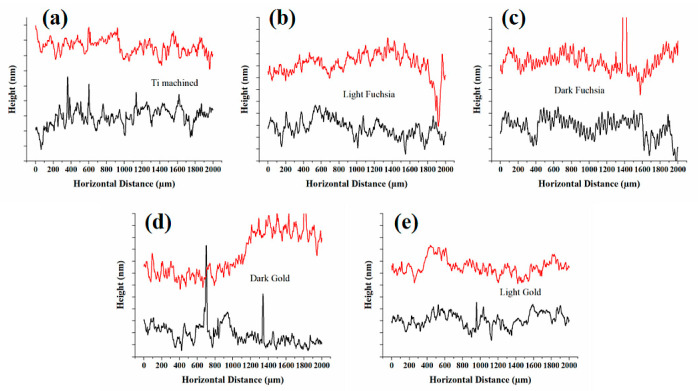
Surface profiles of (**a**) Ti machined, (**b**) light fuchsia, (**c**) dark fuchsia, (**d**) light gold, and (**e**) dark gold discs. Two profiles were reported for any surfaces (red and black lines), where each major vertical tick corresponds to 200 nm.

Ti surfaces were analyzed using a Dektak 6M Surface Profiler to profile surface topography and waviness, as well as measuring surface roughness in the sub-micrometer range. The arithmetic mean roughness (Ra) and the root-mean-square roughness (Rq) over the evaluation length (Table 3). Roughness profiles were reported in Figure 5 (panel a–e). Two different profiles were reported in black and red lines for any surfaces, obtained along the North-South and West-East scanning directions. A tiny contaminant causes a sharp peak in the profile line. The mean roughness values were calculated on at least four profile lines for each surface, and the standard deviation value is equal to ±50 nm for all surfaces.

### 3.5. Scanning Electron Microscopy (SEM)—Energy-Dispersive X-ray Spectroscopy (EDX)

**Figure 6:**
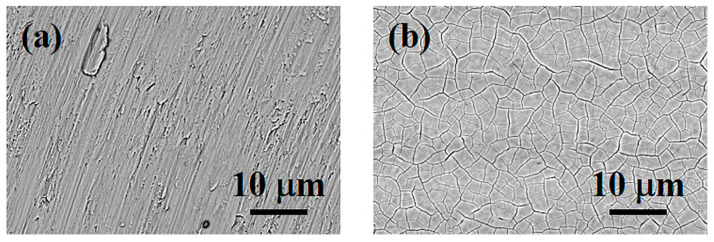
SEM of Ti surfaces: (**a**) machined and (**b**) light fuchsia. Magnification 2000×. Scale bar 10 µm.

The electron scanning microscopy better shows the turning finishing of the surface with rip stack defects, as evidenced in Figure 6, where the machined Ti surface (Figure 6a) and the light fuchsia surface (Figure 6b) are reported. The laser treatment is able to produce a “craquelle-like” surface (Figure 6b) due to the fast cooling of the titanium. Since we are interested in highlighting the colored surfaces of relevant interest for clinical practice and not the influence of all colors, we show only the light fuchsia surface, which is the most promising, and the machined one, taken as a control.

**Figure 7:**
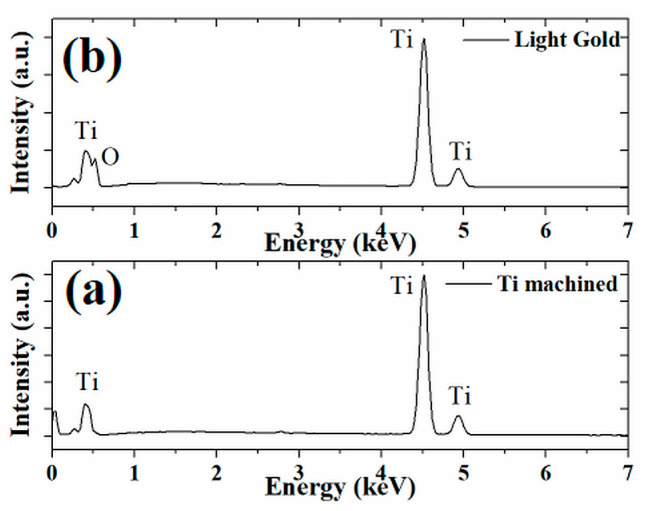
Energy-dispersive X-ray spectroscopy on the surface of (**a**) titanium machined and (**b**) light gold samples.

Energy-dispersive X-ray spectroscopy (EDX) is an analytical technique used for the elemental analysis or chemical characterization of a sample. Figure 7 shows the EDX spectra of the machined Ti surface and the light fuchsia surface. The laser treatment did not produce impurities as confirmed by the EDX measures reported in Figure 7a (only Ti peaks are evidenced for Ti machined surface), but favors the formation of a surface layer of oxide, as shown in Figure 7b. The titanium surface is oxidized through the diffusion of oxygen into the molten metal and the presence of oxygen was verified by SEM/EDX analysis. Analyzing in detail the peaks of the colored sample in Figure 7b, it is possible to notice the presence of oxygen in the spectrum, represented by a resolved peak at about 0.52 keV. The same peak is absent in the worked Ti sample, as shown in Figure 7a.

### 3.6. Viability Evaluation

**Figure 8:**
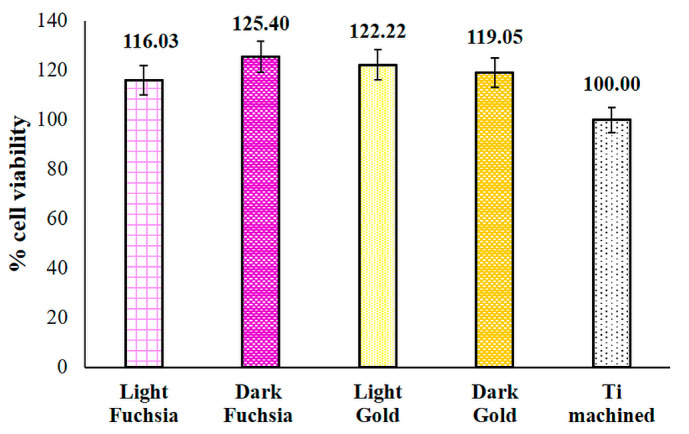
MTT test performed on HGFs cultured for 48 h on titanium surfaces. The most representative of three different experiments is shown. Data are presented as the mean ± standard deviation.

HGF viability was measured after 48 h of culture on selected titanium discs by MTT test. A cell viability increase is recorded in Dark Fuchsia, Light Gold, and Dark Gold surfaces compared to the machined sample (control), even if not statistically significant (Figure 8).

### 3.7. Cytotoxicity Evaluation

**Figure 9:**
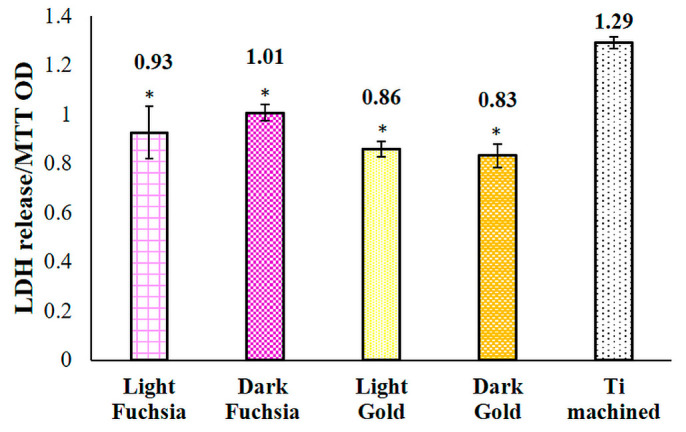
LDH assay of HGFs cultured for 48 h on titanium surfaces. LDH released is reported as OD LDH/OD MTT ratio. Data are presented as the mean ± standard deviation. * vs. CTRL *p* < 0.05.

Cytotoxicity evaluation was carried out by means of LDH assay after 48 h of culture on previously selected titanium discs. The light fuchsia, dark fuchsia, light gold, and dark gold samples all show a statistically significant reduction in LDH release compared to the machined sample (Figure 9).

### 3.8. Collagen I Release

**Figure 10:**
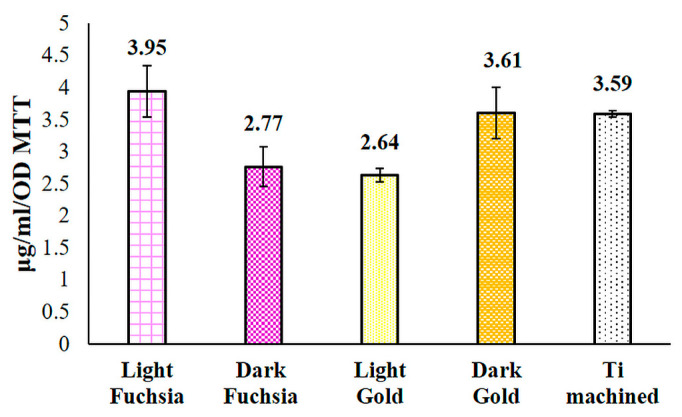
ELISA assay for collagen I secretion in HGFs cultured for 48 h on titanium surfaces. Secretion levels are reported as μg/mL/OD MTT. The bar graph displays densitometric values expressed as mean ± standard deviation.

The release of collagen I within the culture medium was evaluated by means of an ELISA assay after 48 h of culture on previously selected titanium discs. HGFs grown on light fuchsia and dark gold samples show a higher, even if not statistically significant, collagen I release, compared to machined secretion level (Figure 10).

### 3.9. Optical (OM) and Electron Microscopy (SEM) of Laser Colored Surfaces Seeded with HGF

Fibroblast adhesion and growth was observed by stereomicroscope and SEM, on the machined (a) and light fuchsia (b) surfaces. Once again, we show only the light fuchsia surface, which is the most promising, and the machined one, taken as a control.

**Figure 11:**
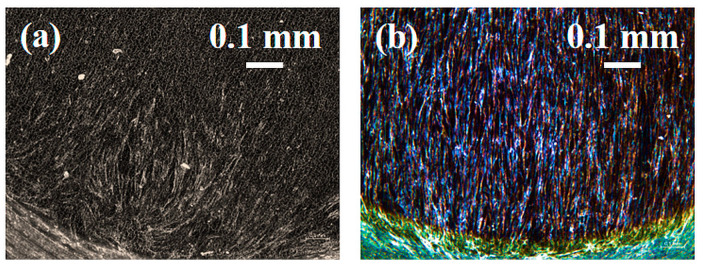
HGF optical images cultured for 48 h on (**a**) Ti machined and (**b**) light fuchsia surfaces by stereomicroscope. Scale bar 0.1 mm.

**Figure 12:**
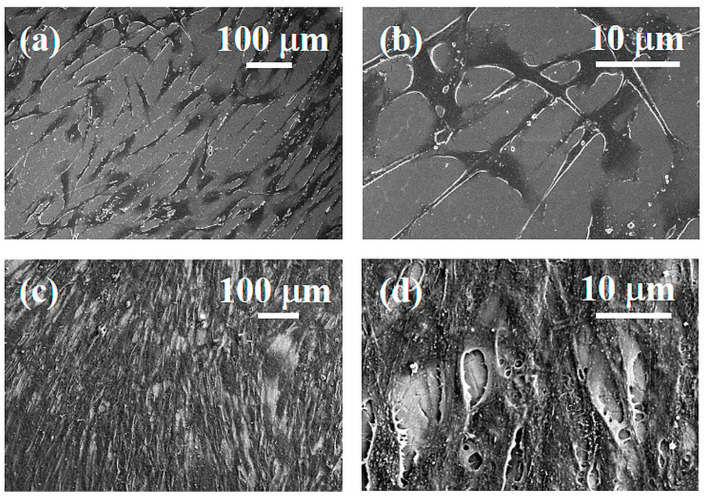
SEM images of HGFs cultured for 48 h on Ti machined surface at 150× (**a**) and 1000× (**b**) magnification, and on light fuchsia surface at 150× (**c**) and 1000× (**d**) magnification.

The stereo microscopy shows that fibroblasts colonization starts from the edges of the specimens (Figure 11). This feature is probably due to the thickness of the specimens. The directions and the orienteering of fibroblasts appear more interesting: in all samples, the circular grooves produced by the lathe rough machining at the edge of the diskettes cause cells to move in circular orientations. At the interface with the colored surface, cells start to tilt their aligning incoming to a direction parallelly aligned with the laser scanning process. After 48 h HGFs of culture, the Optical and SEM analyses at lower magnification showed a small number of cells on the titanium machined surface (Figure 12a). At higher magnification, a small number of “stellate” cells showed thin cell-to-cell connections such as several lamellopodia and filopodia that tightly adhere to the machined surface (Figure 12b). At the same time, it was possible to observe the light fuchsia specimens completely colonized by HGFs (Figure 12c). At higher magnification, a higher number of cells adhered to the surface was observed. Furthermore, a tight network of interconnected cells along with only small areas free from HGFs were also detected (Figure 12d).

## 4. Discussion

Wettability was evaluated by measuring the contact angles of the surfaces with water, and the coloured surfaces showed an increase of contact angle, after laser treatment, with respect to the machined one. All the observed surfaces show a hydrophilic behavior (water contact angle less than 90°), even if it seems that the laser processing decreases the hydrophilic character of the surface itself. It can be noticed that all surfaces have a modest wettability, and wetting is not complete as the measured contact angles are far greater than 0°. Although an increase in the angle was verified, the values obtained are still compatible with those of a modest hydrophilic surface. The order of hydrophilicity of the surfaces is: machined Ti > light fuchsia > dark fuchsia > light gold > dark gold.

By carefully observing the contact angle data, it is possible to divide the series of treated surfaces into two subgroups, which appear to have similar wettability characteristics. The fuchsia surface series is more hydrophilic than the gold ones, but less than the solely machined Ti surface. Since the wetting is the result of surface interactions between a liquid and a solid in contact, it is necessary to measure the surface energies of both liquids and solids to predict wettability. The SFE of a solid surface and its components can provide useful information on how different liquids will interact with the solid surface and is probably the prevailing surface factor influencing cell adhesion strength and proliferation. SFEs with its dispersive and polar components for the Ti surfaces were estimated using the Owens–Wendt–Rabel–Kaelble (OWRK) model [45], by measuring the CA of water (w) and diiodomethane (dim). As observed in Figure 3 and Table 2, the overall SFE of laser-treated Ti surfaces is of the same order of magnitude for all the colored samples, considering the experimental errors, but lower than the machined one.

The small differences between the SFEs are explained by the presence of a different polar component from surface to surface, the dispersive component being almost the same for all laser colored samples. As observed in Figure 3 and Table 2, the overall SFE of laser-treated Ti surfaces is of the same order of magnitude for all the colored samples, considering the experimental errors, but lower than machined one. The polar ratio decreased from 21.5% for the machined Ti surface to 5.7% for the dark gold surface.

The polar component decreased, a trend most evident in the dark gold surface. The laser treatment led to a decrease in the wettability of these surfaces, as the SFE is lowered, and in particular the light gold surface is the one that has less cell adhesion, as demonstrated by collagen I secretion level. Furthermore, in the case of the light fuchsia surface it is possible to observe a much more significant polar contribution compared to the other treated surfaces. It has been previously already reported that cell attachment and spreading are better promoted on surfaces with higher hydrophilic characteristics [48].

Our biological results are in accordance with this assumption and with wettability analyses as they show that HGF adhesion and spreading processes on fuchsia series surfaces appear ameliorated compared to standard machined control. In fact, collagen I secretion and viability percentages, explanatory of adhesion and spreading events, respectively, seem to be enhanced on the aforementioned colored surfaces, which possess, at the same time, the more emphasized hydrophilic/wettable characteristics. Therefore, a positive connection between hydrophilicity of the surface, as well as cell spreading and adhesion, can be established, even if, considering the lack of statistically significant differences among the tested surfaces, further and deepened investigations, including the analysis of a broad panel of adhesion proteins, will be required to confirm this hypothesis. The interfacial energy of a liquid in contact with a solid depends on the dispersive and polar components of the surface energy of the liquid and the solid. From this relationship between the different components, it can be predicted that wettability is generally reduced when the overall surface energy of the solid surface is reduced. For a given liquid with a total surface tension γ_LV_, the contact angle will be minimized if the polar and dispersive ratios of the liquid surface tension match those of the solid SFE. Plotting the polar component, plotted along the *y*-axis against the total surface tension along the *x*-axis (Wetting Envelope, WE), for all the Ti surface can provide a visual investigation of the factors affecting their wettability (Figure 4).

Wetting Envelope plots are particularly useful when studying the effect that the polar and dispersive components of liquid surface tension have on contact angle and wettability. The surface area under the wetting envelope of the machined Ti surface is the largest one. Once again, it is possible to observe a very similar behavior between the two series of colored surfaces, fuchsia and gold, supporting the hypothesis that the wettability is very similar for these two pairs of samples. However, fuchsia surfaces have a larger envelope area than gold surfaces, so they are more wettable. This indicates that the wettability of the latter surfaces is minimal; therefore, the liquid that wets the titanium surface will have a smaller solid–liquid contact area, i.e., less spreading. The polar ratio decreased from 21.5% for the machined Ti surface to 5.7% of the dark gold surface, as reported previously (Table 2). In conclusion, from the contact angle data, the light gold colored surface would appear to be the one in which there is the least spreading of a liquid (such as, for example, a body fluid), therefore the least area of adhesion of the liquid. The light and dark fuchsia surfaces would instead appear to be the ones showing greater wettability. A minor polar component would seem not to assist the adhesion of a liquid to the surface, and this could minimize cell adhesion and proliferation.

However, we must also consider another factor that can influence these phenomena, namely the roughness of the studied surfaces. To highlight the effect of the roughness of these surfaces, measurements were made with a profilometer. In Figure 5, roughness measurements on Ti surfaces are reported. Figure 5a shows the arithmetic mean roughness (Ra) and root mean square roughness (Rq) of the Ti surfaces. The arithmetic mean roughness (Ra) is calculated from the average of the individual heights and depths from the mean elevation of the profile. The root mean square roughness (Rq) is instead calculated from the square root of the sum of the squares of the individual heights and depths. The arithmetic mean roughness values for all surfaces are between 0.15 and 0.3 micron, while the root mean square roughness is between 0.2 and 0.4 micron. The standard deviation values are equal to ± 50 nm for all surfaces. A preliminary analysis of the profiles shows that laser treatment of the titanium surfaces increases the roughness of all observed samples (by about 20–25%), but in the case of the dark gold colored surface, the roughness increase is less pronounced (11%). Several studies have shown that fibroblasts do not attach easily to smooth titanium surfaces and that faster osseointegration has been observed on machined rather than smooth surfaces [21,49,50]. Surface roughness is a factor that can influence not only cell adhesion but also the bacteria adhesion to the titanium surface. Some studies have demonstrated a correlation between the increase in bacterial adhesion, and the consequent formation of biofilm, with surface roughness, and in some cases an arithmetic mean roughness threshold (Ra) of 0.2 µm has been proposed [51,52]. By their nature, bacteria with hydrophobic cell surfaces prefer surfaces of hydrophobic material while those with hydrophilic cell surfaces prefer surfaces of hydrophilic material. Bacterial adhesion increased with higher surface free energies and hydrophobicity of the material. Our laser treated Ti surfaces showed that modest hydrophilicity and biofilm formation should be minimized. Starting from the experimental data, we verified if there could be a correlation between surface parameters and cell viability, cytotoxicity and adhesion. R^2^ values of < 0.3 mean a weak effect on the dependent variable, a value between 0.3 and 0.5 means moderate, and a value > 0.7 means strong effect, i.e., correlation. By analysing in detail the roughness data of the different surfaces in relation to the cellular response, it can be seen that there is not a good correlation between the roughness of the samples and the cellular assays with respective R^2^ below 0.6.

From what was observed with the profilometer, there are no such substantial variations in the roughness of the samples, and this data is confirmed by the biological response.

**Figure 13:**
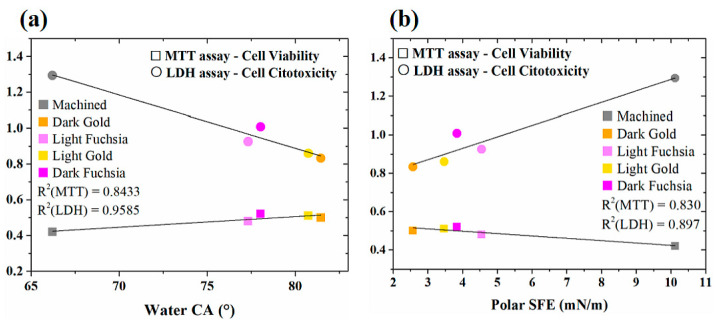
Correlation between (**a**) water CA and (**b**) polar component of SFE of Ti surfaces and cell viability, cytotoxicity, and adhesion.

A strong correlation between water contact angle and MTT/LDH assay was observed (R^2^ = 0.8433 for MTT and R^2^ = 0.9585 for LDH), as reported in Figure 13a. We can assume that a decrease in cell viability/cytotoxicity is observed as the water contact angle of the samples increases, i.e., surfaces become less hydrophilic. No correlation was observed with total SFE or disperse contribution. Only the polar contribution of SFE correlates with MTT/LDH assays (R^2^ = 0.830 for MTT and R^2^ = 0.897 for LDH), as reported in Figure 13b. Cell viability and cytotoxicity decrease with decreasing polar contribution, i.e., a decrease in hydrophilicity and adhesion. As revealed by ELISA essay, the surface that shows the best adhesion is the light fuchsia surface. Light fuchsia is also the most hydrophilic (lower water contact angle), the one with higher polar contribute to SFE (polar ratio 10.4%), and the one with higher wettability (higher area under WE). These results confirm the fact that cell adhesion and proliferation cannot be accurately predicted by a single factor, such as roughness, water Contact Angle values (hydrophilicity), Surface Free Energy (spreading), and Wetting Envelope (wettability). Laser irradiation varies the morphology of the titanium surfaces, increasing their roughness and promoting adhesion, cell proliferation, and viability, as it increases the contact area between implant and bone. The effect of the laser is also to allow the formation of a superficial layer of oxide, which favors the wettability of body fluids and consequently the compatibility with blood, which leads to better osseointegration. The laser treatment, therefore, has the multiple function of improving the interaction between bone and implant, but also the possibility of obtaining a colored implant, which blends better with the color of the regrowth dental gum, compared to the gray machined Ti.

## 5. Conclusions

The present study demonstrated that the technique used to obtain the titanium surfaces colored with laser ytterbium has the advantage of having produced, in an ecological way, surfaces that are all biocompatible with HGFs. This was especially confirmed by LDH assay, highlighting that the recorded cytotoxicity on all tested surfaces is reduced compared to the uncolored one (machined). Furthermore, cell viability is very high on all studied surfaces, as confirmed by both MTT test and by SEM analysis, which clearly evidences a dense cell network, despite these surfaces showing a modest hydrophilic behavior, and do therefore not become too wet in contact with an aqueous fluid such as those of the body. The laser treatment has led to a decrease in the wettability of the surfaces, as the SFE is lowered for all the colored surfaces compared to the machined one, but the decrease in wettability did not affect HGF growth. Thanks to the lathe rough machining, fibroblasts start to proliferate in oriented directions at the edge of the disks, aligned parallelly to the laser grooves. Laser treatment of the titanium surfaces increases the roughness of all observed samples, compared to the machined one, amplifying this beneficial effect towards cell growth.

Although all the colored surfaces show excellent biocompatibility and a very high rate of cell viability, light fuchsia appears to be the more promising as it further guarantees, on one hand, a higher collagen I secretion, which could be predictive of an ameliorated cell adhesion, and, on the other hand, a higher hydrophilicity (lower water contact angle), polar contribute to SFE (higher polar ratio), and wettability (higher area under WE).

In addition, from a purely aesthetic point of view, the choice of a fuchsia-colored surface in a dental implant is the most suitable to obtain color integration with the gums.

All in all, the obtained results underline that the best performance was evidenced for the light fuchsia colored Ti surface in terms of biocompatibility, cell viability, and adhesion, but also from an aesthetic point of view.

Therefore, the laser coloring methods adopted can be properly used for aesthetic, colored dental implants with gingival aesthetic features that are highly replicable, contaminant free, eco-friendly, and cheap. Further and extensive in vivo studies will be required to increase the statistical significance of all the data, and to clarify the molecular mechanisms underlying the biological processes, such as cell adhesion, proliferation, and cytotoxicity, on which the authors focused in this paper. However, the present study can help dental clinicians and oral implantologists to evaluate the impact of titanium surface modifications on the color of titanium implants and abutments. The ytterbium laser treatment of the titanium surfaces is biocompatible and allows for the creation of a complete color palette while maintaining the roughness of the titanium surface and it is able to promote the growth of soft tissues in all the evaluated samples with some interesting aesthetic differences without significance statistics.

## Figures and Tables

**Table 1:** Chromatic coordinates, expressed in the L*a*b* system, of Ti surfaces machined, light fuchsia, dark fuchsia, light gold, dark gold, and color variation (∆E) compared with the machined titanium surface.

Sample	L*	a*	b*	∆E
Machined	68.49	−0.40	−0.91	-
Light Fuchsia	27.49	+41.04	+55.21	48.91
Dark Fuchsia	17.42	+40.34	+50.31	38.84
Light Gold	88.12	−07.32	+34.05	25.33
Dark Gold	50.02	+09.32	+52.63	28.58

**Table 2:** Water and diiodomethane Contact Angle data for coloured Ti discs. Surface free energy is also reported, together with dispersive and polar contributes, and polar ratio.

Sample	Water CA (°)	Diiodo Methane CA (°)	SFE (mN/m)	SFE Disperse (mN/m)	SFE Polar (mN/m)	Polar Ratio (%)
Ti machined	66.21 (±3.68)	45.16 (± 2.92)	47.04 (±3.55)	36.93 (±1.56)	10.12 (±1.98)	21.5
Light Fuchsia	77.32 (±9.44)	41.19 (±2.07)	43.56 (±4.56)	39.01 (±1.06)	4.55 (±3.5)	10.4
Dark Fuchsia	78.03 (±12.26)	36.88 (±1.48)	44.99 (± 4.9)	41.14 (±0.71)	3.84 (±4.19)	8.5
Light Gold	80.74 (±5.91)	42.23 (±1.36)	41.94 (±2.65)	38.47 (±0.71)	3.47 (±1.94)	8.3
Dark Gold	81.45 (±0.02)	34.41 (±0.02)	44.87 (±0.02)	42.30 (±0.01)	2.57 (±0.01)	5.7

**Table 3:** Roughness measurements on Ti discs. The arithmetic mean roughness (Ra) and the root-mean-square roughness (Rq) of the Ti surfaces were reported. The standard deviation value is equal to ± 50 nm for all surfaces.

Sample	Ra (nm)	Rq (nm)
Ti machined	191 (±50)	247 (±50)
Light Fuchsia	254 (±50)	311 (±50)
Dark Fuchsia	259 (±50)	324 (±50)
Light Gold	254 (±50)	315 (±50)
Dark Gold	215 (±50)	278 (±50)

## Data Availability

No new data were created or analyzed in this study. Data sharing is not applicable to this article.

## Sample Availability

Samples of the Titanium discs are available from the authors.
